# Supplementary material for: Electrocrystallization of Calcium Oxalate on Electrospun PCL Fibers Loaded with Phytic Acid as a Template
Source: Polymers (Basel). 2022 Aug 5;14(15):3190. doi: 10.3390/polym14153190 (PMC9371010; doi:10.3390/polym14153190)
Supplement: Supplementary file 1 [file polymers-14-03190-s001.zip › polymers-1834714-supplementary.pdf]

Electrocrystallization of Calcium Oxalate on the Electrospun PCL Fibers Loaded with Phytic Acid as Template

Tatiana Zegers Arce [1], Mehrdad Yazdani-Pedram [2], and Andrónico Neira-Carrillo [1,*]

1. Department of Biological and Animal Sciences, Faculty of Veterinary and Animal Sciences, University of Chile, Santiago, Santa Rosa 11735. La Pintana, 8820808, Santiago, Chile; tatiana.zegers.a@gmail.com (T.Z.A.)
2. Department of Organic and Physical Chemistry, University of Chile. S. Olivos 1007. Independencia, 8380544, Santiago, Chile; myazdani@ciq.uchile.cl (M.Y.-P)

* Correspondence: aneira@uchile.cl; Tel.: +562-29785674

Supplementary Materials

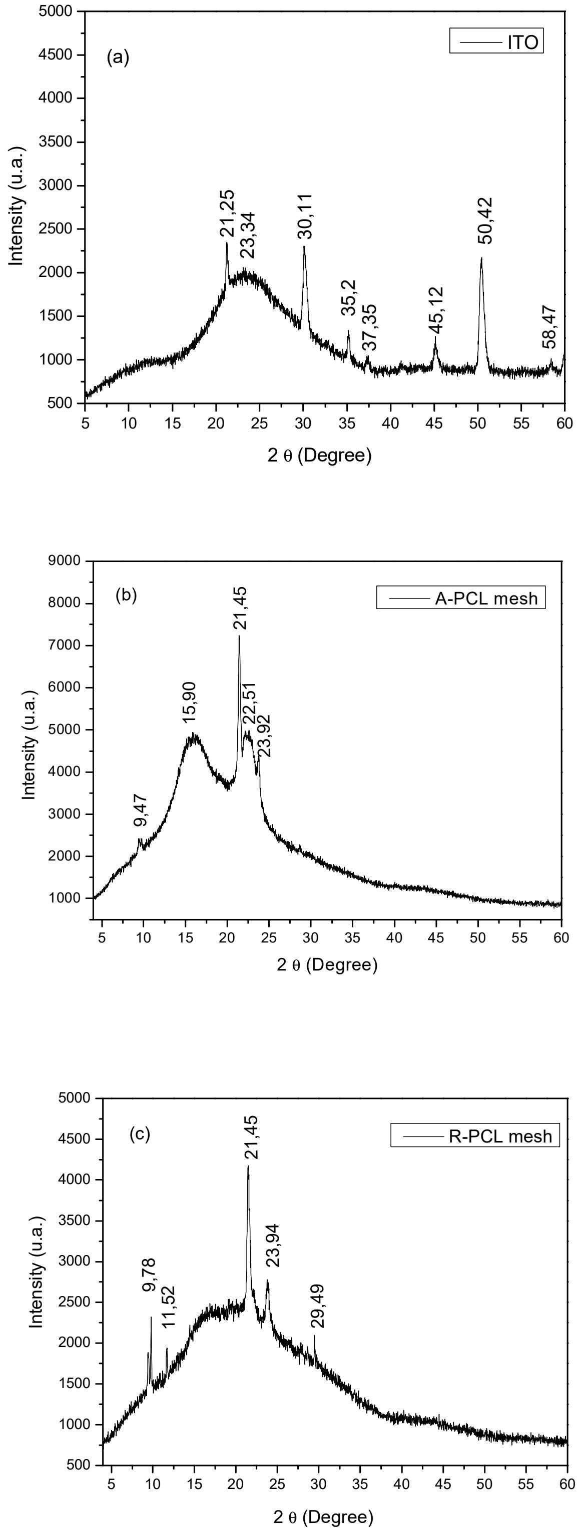

Figure S1. XRD diffractograms of ITO glass substrate and controlled PCL-ESM meshes utilized as controls: (a) ITO, (b) A-PCL, and (c) R-PCL.

Table S1: Crystal number determination, average number of CaOx crystal number estimation (crystal numbers/mm2), confidence interval and statistical considerations in the CaOx EC assays using Minitab 19.

Sample	Total number of crystals (N)	Crystals/mm^2	Number of crystals (n)	Average size (µm)	Standard deviation	Confidence interval 95%
CB	310	3.192	172	12,965	8,341	(11,709; 14,220)
CN-A	150	2.005	108	7,933	8,518	(6,308; 9,558)
CN-R	42	368	38	15,70	8,92	(12,77; 18,63)
PA-A 1 mg/l	451	5.098	208	5,598	3,759	(5,084; 6,111)
PA-R 1 mg/l	142	1.484	109	4,445	2,565	(3,958; 4,932)
PA-A 1,5 mg/l	133	1.596	99	20,64	15,01	(17,65; 23,64)
PA-R 1,5 mg/l	276	2.897	161	9,578	7,890	(8,350; 10,806)

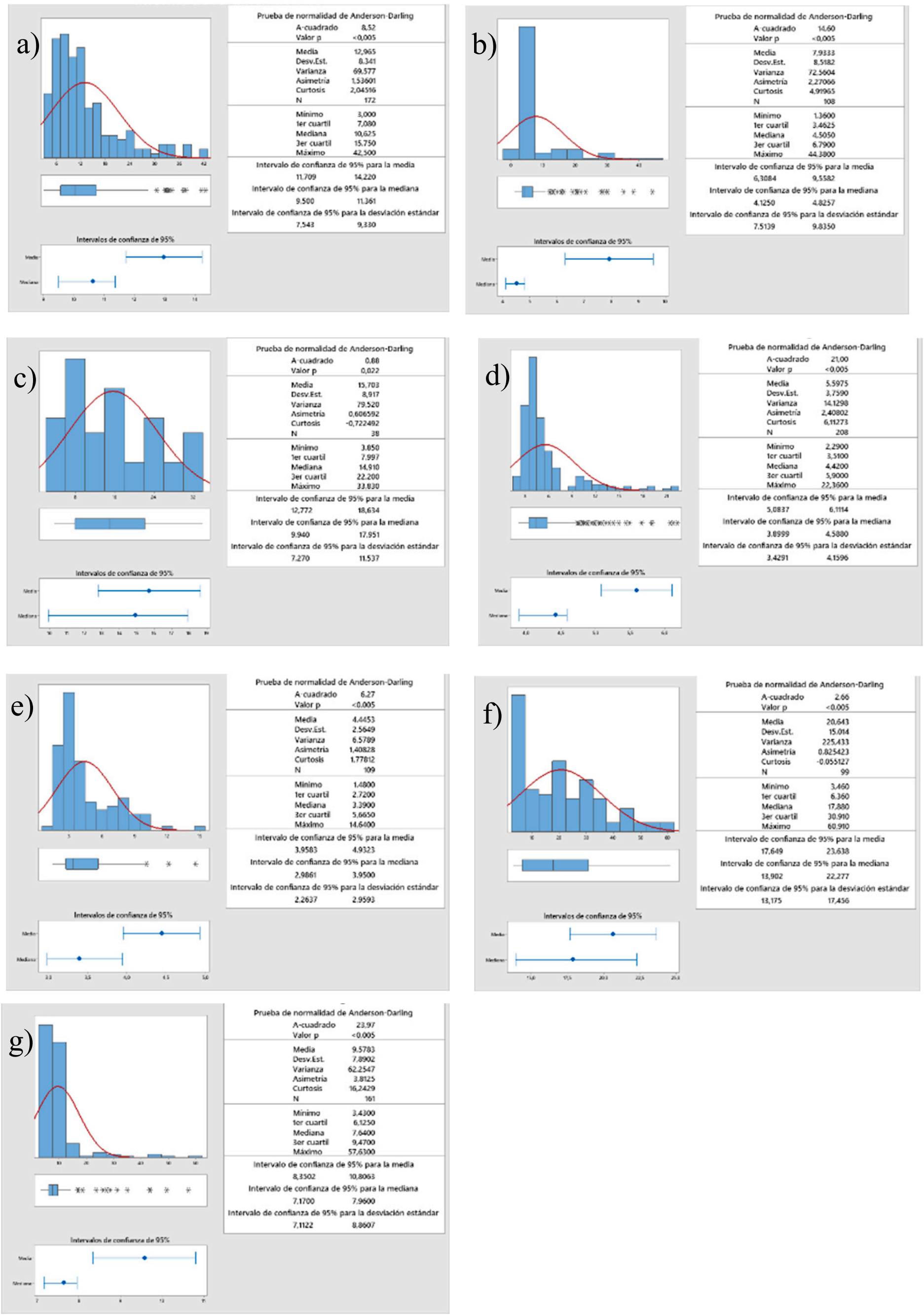

Figure S2: Normality test (Anderson-Darling). Report summaries. a) 1 BC, b) 2 CN-A, c) CN-R, d) 4 PA-A, e) 5-PA-R, f) 6 PA-A, g) 7 PA-R

Method

Null hypothesis	All variances are equal
Alternative Hypothesis	At least one variance is different
Significance Level	α = 0,05

Bonferroni confidence intervals (95%) for standard deviations

Sample	n	SD	CI
1 CB	172	8,3413	(6,6442; 10,6493)
2 CN-A	108	8,5182	(5,7756; 12,9057)
3 CN-R	38	8,9174	(6,7979; 12,6517)
4 PA-A 1 mg	208	3,7590	(2,7934; 5,1290)
5 PA-R 1 mg	109	2,5649	(1,9452; 3,4734)
6 PA-A 1,5 mg	99	15,0144	(12,2203; 18,9973)
7 PA-R 1,5 mg	161	7,8902	(4,7451; 13,3574)

Individual confidence level = 99,5833%

test

Method	Test stadistics	P -value
Multiple comparisons	—	0,000
Levene	49,51	0,000

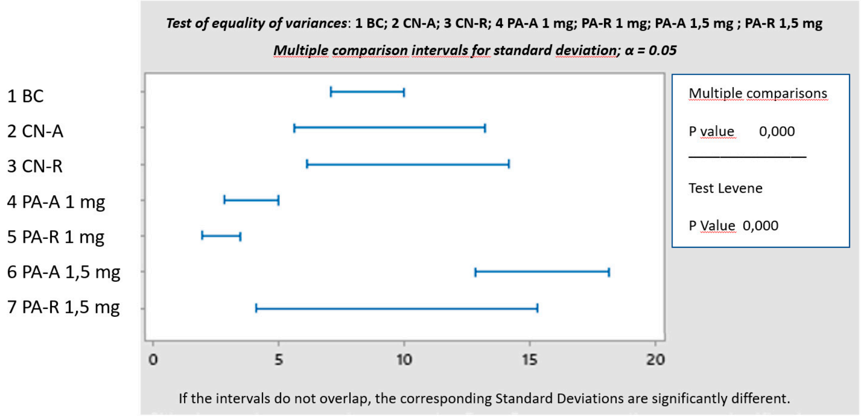

Figure S3. Equality of variances test (Bonferroni - Levene).

Method

Null hypothesis	All means are equal
Alternative Hypothesis	Not all means are equal
Significance level	α = 0,05

Equal variances were not assumed for the analysis.

A) Median

Factor	n	Median	SD	CI of 95%
2 CN-A	108	7,933	8,518	(6,308; 9,558)
4 PA-A 1 mg	208	5,598	3,759	(5,084; 6,111)
6 PA-A 1,5 mg	99	20,64	15,01	(17,65; 23,64)

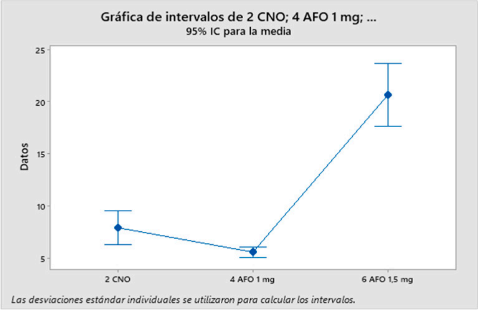

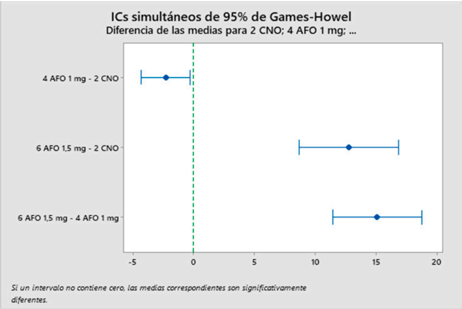

B) Median

Factor	N	Median	SD	CI of 95%
3 CN-R	38	15,70	8,92	(12,77; 18,63)
5 PA-R 1 mg	109	4,445	2,565	(3,958; 4,932)
7 PA-R 1,5 mg	161	9,578	7,890	(8,350; 10,806)

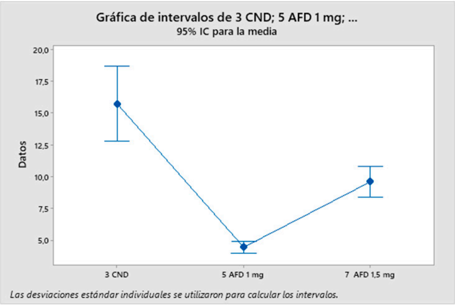

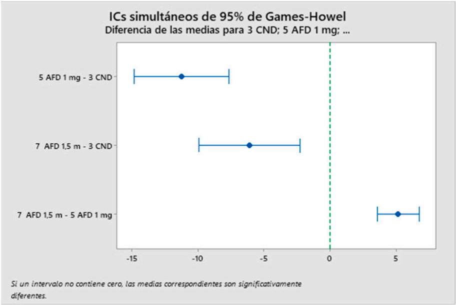

C) Median

Factor	N	Median	SD	CI of 95%
1 BC	172	12,965	8,341	(11,709; 14,220)
2 CN-A	108	7,933	8,518	(6,308; 9,558)
3 CN-R	38	15,70	8,92	(12,77; 18,63)
4 PA-A 1 mg	208	5,598	3,759	(5,084; 6,111)
5 PA-R 1 mg	109	4,445	2,565	(3,958; 4,932)
6 AFO 1,5 mg	99	20,64	15,01	(17,65; 23,64)
7 AFD 1,5 mg	161	9,578	7,890	(8,350; 10,806)

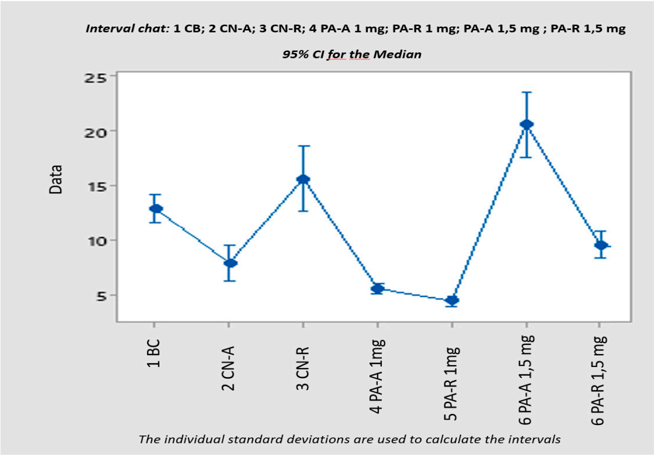

Figure S4. Welch's test - Paired comparison Games Howell. Analysis A - C.

*Note * It is not possible to draw the interval plot for the Games-Howell procedure. Interval plots for comparisons are unreadable with more than 45 intervals.*
